# *In Vitro* Dedifferentiation of Melanocytes from Adult Epidermis

**DOI:** 10.1371/journal.pone.0017197

**Published:** 2011-02-23

**Authors:** Bernadett Kormos, Nóra Belső, Attila Bebes, Gábor Szabad, Sarolta Bacsa, Márta Széll, Lajos Kemény, Zsuzsanna Bata-Csörgő

**Affiliations:** 1 Dermatological Research Group of the Hungarian Academy of Sciences, Szeged, Hungary; 2 Department of Dermatology and Allergology, Faculty of Medicine, University of Szeged, Szeged, Hungary; Biological Research Center of the Hungarian Academy of Sciences, Hungary

## Abstract

In previous work we described a novel culture technique using a cholera toxin and PMA-free medium (Mel-mix) for obtaining pure melanocyte cultures from human adult epidermis. In Mel-mix medium the cultured melanocytes are bipolar, unpigmented and highly proliferative. Further characterization of the cultured melanocytes revealed the disappearance of c-Kit and TRP-1 and induction of nestin expression, indicating that melanocytes dedifferentiated in this *in vitro* culture. Cholera toxin and PMA were able to induce c-Kit and TRP-1 protein expressions in the cells, reversing dedifferentiation. TRP-1 mRNA expression was induced in dedifferentiated melanocytes by UV-B irradiated keratinocyte supernatants, however direct UV-B irradiation of the cells resulted in further decrease of TRP-1 mRNA expression. These dedifferentiated, easily accessible cultured melanocytes provide a good model for studying melanocyte differentiation and possibly transdifferentiation. Because melanocytes in Mel-mix medium can be cultured with human serum as the only supplement, this culture system is also suitable for autologous cell transplantation.

## Introduction

In vertebrates, melanocytes arise from the neural crest [1]. Melanoblasts are unpigmented cells containing only immature melanosomes that lack functional tyrosinase, the critical enzyme of melanin synthesis [2]. Fully differentiated melanocytes characterized by tyrosinase (TYR), tyrosinase related protein-1 and -2 (TRP-1 and TRP-2) activities as well as by numerous mature melanosomes and well-developed dendrites, are located mainly in the epidermis, dermis and hair bulb [3]. Examination of melanoblasts is important to analyze basic mechanisms of cell differentiation, and to study the pathomechanisms of melanoma and genetic disorders of melanocyte development [4].

Melanocyte differentiation is under the control of microphthalmia transcription factor (MITF), a basic helix-loop-helix leucine zipper transcription factor, that activates genes involved in pigment production, such as TYR, TRP-1 and TRP-2[5] and melanocyte survival, e.g. Bcl-2 [5], [6]. Ectopic expression of MITF in embryonic fibroblast induces growth inhibition and morphologic changes consistent with melanocyte differentiation [7]. The transcriptional activity of MITF is modified by mitogen-activated protein kinase (MAPK) phosphorylation [8]. Kit is essential for melanocyte migration, survival, proliferation and differentiation [9]. In mouse epidermis, Kit^+^ cells differentiate into Mitf^+^ and/or TRP-2^+^ cells first and then into TRP-1^+^ cells after UV exposure [10]. In human skin, the presence of Kit reactive cells is consistently demonstrated in the basal layer of the epidermis, follicular infundibula and eccrine coils and ducts. In the follicular infundibula, Kit^+^Bcl-2^+^TRP-1^–^ cells represent a reserve population of precursor melanocytes [11]. In mouse, towards the completion of hair follicle morphogenesis, several distinct follicular melanocytic cell populations were defined: undifferentiated, non-pigmented c-Kit-negative melanoblasts in the outer root sheath and bulge and highly differentiated melanocytes adjacent to the hair follicle dermal papilla above the Auber's line [12]. Interestingly, autocrine SCF stimulation of Kit receptor seems to be an important step in melanoma genesis in its early phases, but it is down-regulated in later stages [13], [14].

The first reproducible melanocyte culture technique was established in 1982 [15]. Generally, melanocyte culturing *in vitro* is accomplished by using specific mitogens to enhance melanocyte and to suppress keratinocyte and fibroblast growth. Usually the melanocyte culture media is supplemented with the tumor promoter 12-*O*-tetradecanoyl phorbol-13-acetate (TPA or PMA) and the intracellular cyclic adenosine 3′, 5′ monophosphate (cAMP) enhancer, cholera toxin (CT) and in some cases 3-isobutyl-1-methylxanthine (IBMX) is used [16]. Because these mitogens alter the physiological responses of the cells, attempts have been made to define culturing techniques free of these substances [17]–[19]. Earlier we have shown that normal human adult epidermal melanocytes can grow in a medium (referred to as Mel-mix) that lacked the above listed mitogens. In Mel-mix medium melanocytes proliferate rapidly, they become bipolar and they lose their pigmentation after a few weeks in culture. Cell proliferation slows between the 10^th^ and 15^th^ passages and cells reach complete senescence around passage 15 [20].

In the present work we show that melanocytes dedifferentiate in this cholera toxin and PMA-free culture. During this dedifferentiation process c-Kit and TRP-1 expressions decrease in the cells, their expression of epidermal growth factor receptor (EGFR) and nestin proteins increase, as well as their proliferative capacity. We also show that *in vitro* dedifferentiation in the culture is a reversible process, both CT and PMA treatment and UVB irradiation can induce *in vitro* redifferentiation of melanocytes. The culture technique developed in our laboratory provides a good model system to study mechanisms of cellular differentiation of normal human epidermal melanocytes. Further advantage of this culture method is that these melanocytes are applicable for transplantation.

## Materials and Methods

### Culture media

Mel-mix medium contains AIM-V serum free lymphocyte medium and Keratinocyte Serum Free Medium (both from Life Technologies, Carlsbad, CA, USA), v∶v, supplemented with 2.5% fetal bovine serum (FBS, Life Technologies), 2.5 ng/ml epidermal growth factor (EGF, Life Technologies), 25 µg/ml bovine pituitary extract (BPE, Life Technologies), L-glutamine and Antibiotic Antimycotic Solution containing 100 U/ml penicillin, 100 µg/ml streptomycin and 0.25 µg/ml amphotericin B (Sigma Laboratories, St. Louis, MO, USA).

We also used a commercially available melanocyte medium, M254 Medium (Life Technologies). This medium is supplemented with Human Melanocyte Growth Supplement (HMGS, Life Technologies) containing bovine pituitary extract (BPE), fetal bovine serum, bovine insulin, bovine transferrin, basic fibroblast growth factor, hydrocortisone, heparin and phorbol 12-myristate 13-acetate (PMA).

To induce melanocyte differentiation, the Mel-mix medium was supplemented with cholera toxin (CT, Sigma) at 10 nM and phorbol 12-myristate 13-acetate (PMA, Sigma) at 10 ng/ml concentration, respectively.

### Cell culture

Adult epidermal melanocytes were isolated and cultured as previously described [20] from breast or trunk skin specimens of healthy Caucasian donors undergoing plastic surgery. The study was approved by the Human Investigation Review Board of the University of Szeged: it complied with the ethical standards of research, in accordance with the Helsinki Declaration. Written informed consent was obtained from all donors involved in the study.

Skin specimens were first washed in Salsol A solution (Human Rt, Godollo, Hungary) supplemented with 2% Antibiotic Antimycotic Solution (Sigma). The subcutis and part of the dermis was removed and the tissue was cut into small strips. Overnight incubation in Dispase solution (Grade II, Roche Diagnostics, Mannheim, Germany) was carried out at 4°C to separate the dermis from the epidermis. To prove that the use of dispase prevents fibroblast contamination, we isolated RNA both from the epidermis and the dermis after dispase treatment and performed real-time RT-PCR using primers specific for COL1A2, the gene that encodes for the alpha 2 chain of type I collagen. We could not detect COL1A2 gene expression in the epidermal samples (Figure S1) [20]. Thus, the use of dispase prevents fibroblast contamination in the epidermal cell cultures. Next day the epidermis was peeled of the dermis. The epidermis was put into 0.25% trypsin (Sigma) for 30 minutes at 37°C. Following trypsinization, the epidermis was mechanically torn apart and vigorously washed to release epidermal cells. The epidermal cell suspension was filtered through a 100 µm nylon mesh (BD Falcon, San Jose, CA, USA) and centrifuged at 200 *g* for 10 minutes at 4°C. Epidermal cells were then placed into 75 cm^2^ tissue culture dishes (BD Falcon) at a cell density of 2×10^5^ cells/cm^2^. In Mel-mix medium at the beginning of the culture melanocytes usually attach in 24 hours, while keratinocytes can attach later between 24–48 hours after plating. We therefore wash the plates between 12–24 hours after plating to remove keratinocytes and supply fresh Mel-mix medium to the cells. If this was not enough to prevent our culture from keratinocyte contamination, at the first passage keratinocytes are completely removed from the cultures by short trypsinization with 0.01% Trypsin/EDTA, Sigma. Due to the different attachment characteristics of keratinocytes and melanocytes to the culture plastic, melanocytes release 2–3 minutes earlier than keratinocytes, thus enabling separation of the two cell populations (Figure S2). Fresh culture media was put on cells three times weekly. Primary melanocyte cultures reached ∼90% confluency in 7–9 days. Confluent primary cultures were treated with 0.05% EDTA in PBS and cells were harvested by a short, 2–3 minutes trypsinization. Harvested cells were divided into two equal parts at passages. Cultures were grown at 37°C in humidified atmosphere containing 5% CO_2_ [20].

Keratinocytes prepared from similar epidermal samples were cultured in KSF medium (Life Technologies) as described previously [21].

### Real-Time RT-PCR

Total RNA was isolated from 1×10^6^ cultured melanocytes by TRIZOL™ reagent (Life Technologies) following the instructions of the manufacturer. RNA concentrations were determined by the A_260_ values of the samples. Complementary DNA (cDNA) was synthesized from 1 µg of total RNA using iScript cDNA Synthesis Kit (Bio-Rad, Hercules, CA, USA). RT-PCR was used to quantify the relative abundance of each mRNA (iCycler IQ Real Time PCR, BioRad, Hercules, CA, USA). After reverse transcription, amplification was carried out using FastStart TaqMan Probe Master from Roche (Basel, Switzerland). Primers were as follows: TRP-1 forward: CTTTTCTCACATGGCACAGG, TRP-1 reverse: AAGGCTCTTGCAACATTTCC, probe: #10 from the Roche Universal ProbeLibrary, (cat. no. 04685091001); c-Kit forward: CGTGGAAAAGAGAAAACAGTCA, c-Kit reverse: CACCGTGATGCCAGCTATTA, c-Kit probe: #2 from the Roche Universal ProbeLibrary, (cat. no. 04684982001); nestin forward: TGCGGGCTACTGAAAAGTTC, nestin reverse: TGTAGGCCCTGTTTCTCCTG, nestin probe: #76 (cat. no. 04688996001); 18S RNA forward: CTCAACACGGGAAACCTCAC, 18S RNA reverse: CGCTCCACCAACTAAGAACG, 18S probe: #77 from the Roche Universal ProbeLibrary, (cat. no. 04689003001). The amplification protocol for TRP-1 and c-Kit expression contained one cycle of initial denaturation at 95°C for 3 min followed by 40 cycles of denaturation at 95°C for 15 sec and annealing/extension at 57°C for 1 min. The same protocol with 25 cycles was applied for the detection of 18S mRNA expression.

### Immunocytochemistry

For cytospin preparation cells were harvested by trypsinization. After washing, the cell pellet was resuspended in PBS and the cell density was set at 1×10^6^ cells/ml. 100 µl cell suspension was put into plastic tubes and centrifuged (Cytopro, Wescor, Logan, Utah, USA) onto glass slides, then dried overnight at 25°C. Air-dried slides were fixed in 2% paraformaldehyde (Sigma) for 20 minutes. Slides were incubated with primary antibodies specific for TRP-1 at a dilution of 1∶2000 (Signet Laboratories, Dedham, MA, USA) for c-Kit (BD) at a dilution of 1∶250 and for EGFR (Clone: sc-120, Santa Cruz Biotechnologies, Santa Cruz, CA, USA) at a dilution of 1∶200 in a staining solution containing TBST (Tris buffered saline containing 0.1% Triton-X) (Sigma) and 0.5% BSA (Sigma) for 1 hour. Control slides were incubated with mouse IgG_2a_ (Sigma), the isotype of all primary antibodies. Slides were then incubated with a biotinylated secondary antibody (Vectastain ABC Kit, Vector, Burlingame, USA) for 1 hour at room temperature, followed by the blocking of endogen peroxidase with 1% H_2_O_2_ (Sigma) in methanol (Spektrum 3D, Debrecen, Hungary) for 20 minutes. After that the slides were incubated with horse radish peroxidase-conjugated streptavidin for 1 hour at room temperature (Vectastain ABC Kit, Vector). The peroxidase activity was visualized using 3-amino 9-ethylcarbazole (AEC, Sigma) as a substrate. Finally, the slides were counterstained with Mayer's hematoxylin (Sigma). The cells were analyzed using a Zeiss Axio Imager microscope and photographed using a PixeLINK digital camera (TissueGnostics, Austria). For quantification of the positive cells, the Cell Counter option of ImageJ software (freely available program from NIH) was used.

### Western blot

Cells were trypsinized and harvested by centrifugation, and the pellet was then gently resuspended in protein lysis buffer (20 mM HEPES, 150 mM KCl, 1 mM MgCl_2_, 1 mM dithiothreitol, 10% glycerol, 0.5% Triton X-100, 0.1% Igepal® CA-630) containing 0.5% protease inhibitor cocktail (all components from Sigma). Protein concentrations were determined with the BCA detection kit (Thermo Scientific, Waltham, MA, USA). SDS-PAGE was carried out with 40 µg protein samples, blotted to a nitrocellulose membrane (Bio-Rad). Membranes were blocked in Tris-buffered saline (TBS, 150 mM NaCl, 25 mM Tris, pH 7.4) containing 3% non-fat dry milk powder (Bio-Rad). Mouse anti-human nestin (Abcam) was used at 1 µg/ml concentration and rabbit anti-human α-actin (Sigma) was diluted at 1∶400 and incubated the nitrocellulose membrane with them overnight at 4°C. Anti-rabbit and anti-mouse IgG alkaline phosphatase-conjugated secondary antibodies (Sigma) were applied and the bands were visualized using SigmaFAST BCIP/NBT (Sigma).

### Direct and indirect UVB irradiation

For UVB irradiation, a VL-6LM light source (Vilber Lourmat, Marne-la-Vallée, France) was used. The light emitted from this lamp was within the UVB range (280–320 nm) and the peak emission was at 312 nm. Light intensity was measured by a UVX radiometer (UVP, Upland, CA, USA) before the experiments. To study the direct effect of UVB, 3^rd^ passage, Mel-mix-cultured normal human adult epidermal melanocytes in PBS were irradiated with 0, 20.8, 31.2 and 41.6 mJ/cm^2^ doses of UVB. After irradiation, prewarmed medium (37°C) was put on the cells. To examine the indirect effects of UVB, cultured normal human adult epidermal keratinocytes in PBS were irradiated with the same doses of UVB and supernatants of the irradiated keratinocytes were collected 6 and 24 hours after irradiation. These supernatants were mixed with AIM-V medium (v∶v) supplemented with 5% FBS (Hyclone, Logan, UT, USA), 1% L-glutamine and 1% Antibiotic Antimycotic Solution and autologous cultured melanocytes in 3^rd^ passage were cultured in them for 24 hours, then total RNA was isolated from the cells. Cell viability was measured during the experiment, these data are included in the Results.

### Carboxyfluorescein diacetate, succinimidyl ester (CFSE) analysis

For CFSE analysis, CellTrace CFSE Cell Proliferation Kit (Life Technologies) was used according to the manufacturer's instructions. Cells were trypsinized, washed and resuspended in PBS. Stock CFSE was added to the cell suspension at 0.5 µM final concentration. The suspension was incubated for 15 minutes at 37°C. After incubation, cells were centrifuged and resuspended in fresh prewarmed medium (37°C), then plated and cultured for 96 hours. For control culture (0-hour sample) cells were incubated at 37°C in suspension with fresh medium for 30 minutes. CFSE-labeled cells were trypsinized and measured by flow cytometry in every 24 hours between 0 to 96 hours. After a washing procedure, CFSE fluorescence in the FL-1 channel was measured using a dual-laser FACS-Calibur flow cytometer (Beckton Dickinson, San Jose, CA) and analyzed with CellQuest Software. The number of cell divisions was calculated based on the assumption that the dye of the mother cells would be equally divided into both daughter cells, resulting in halving of fluorescence intensity.

### Senescence-associated β-galactosidase assay

To determine the senescent stage of the cells, 3^rd^ and 8^th^ passage melanocytes cultured in Mel-mix or in M254 medium were stained using Senescence-associated β-galactosidase Staining Kit (Cell Signaling Technology, Danvers, USA) according to the manufacturer's instructions. Briefly, cells were fixed in 2% paraformaldehide for 15 minutes at room temperature, washed, then incubated with X-gal (5-bromo-4-chloro-3-indolyl-b-D-galactopyranoside) at 1 mg/ml concentration in a special staining solution overnight at 37°C. Next day, the cells were mounted with 70% glycerol and were visualized using a Nikon Eclipse microscope and photographed by a Nikon camera. For quantification of the positive cells, the Cell Counter option of ImageJ software (freely available program from NIH) was used.

### Data presentation and statistical analysis

PCR results are expressed as fold increases over control values. Data are presented as mean ± standard deviation (SD). Data were compared using the one-way analysis of variance (Univariate ANOVA) followed by Tukey's and Dunnett's *post hoc* test to determine statistical differences after multiple comparisons (SPSS, SPSS, Chicago, Illinois). A probability value of less than 0.05 was considered significant.

## Results

### TRP-1 and c-Kit expressions decrease in human adult melanocytes cultured in Mel-mix medium

**Figure 1:**
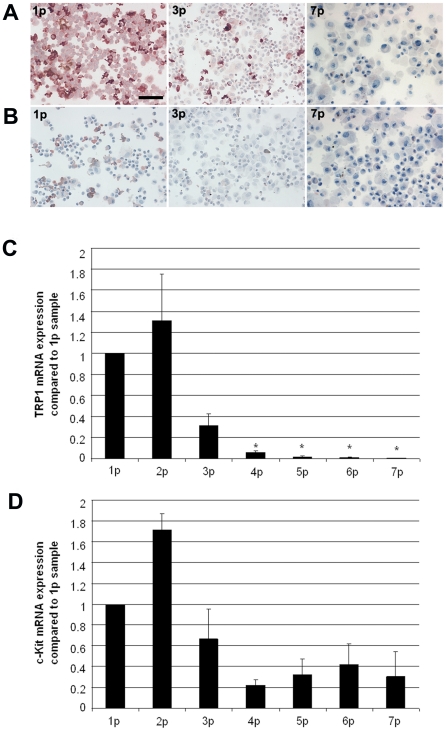
TRP-1 and c-Kit expressions decrease in human melanocytes cultured in Mel-mix medium. Both TRP-1 (A) and c-Kit (B) protein expressions were present in primary cultures, 100% of cells expressed TRP-1 and 30% expressed c-Kit (1p), the expression of both decreased during *in vitro* culturing. Melanocytes in 7^th^ passage (7p) expressed neither TRP-1 nor c-Kit proteins. Bar: 100 µm. TRP-1 and c-Kit mRNA expressions were detected in cultured melanocytes at every passage by real-time RT-PCR analysis. TRP-1 (C) mRNA expression decreased significantly, while c-Kit (D) mRNA expressions showed a decreasing trend during *in vitro* culturing. Values are shown as relative expressions compared to the first passage (1p) sample (mean±SD from three independent experiments; *significant at p≤0.05, Univariate ANOVA).

Cytospins were prepared from melanocyte cultures at an early phase (first passage in culture, approximately 7–10-day-old culture), at 3^rd^ passage in culture (3p, about three weeks after primary plating) and at late phase in the 7^th^ passage cultures (generally five weeks into culture). TRP-1 protein expression was detected using a monoclonal antibody (Mel-5). TRP-1 exhibited strong expression in primary melanocyte cultures (100% of the cells were positive), and its expression decreased (33% in 3^rd^, 0% in 7^th^ passage culture) during *in vitro* culturing in Mel-mix medium (Figure 1A). c-Kit protein expression was also strong in 30% of the cells in the early cultures (1^st^ and 2^nd^ passages), but melanocytes never stained as uniformly with the anti-c-Kit antibody as with the Mel-5 antibody (Figure 1B). In 3^rd^ passage cultures only 4% of the cells were positive for c-Kit protein (Figure 1B) and all cells were TRP-1 and c-Kit negative in the five-week-old, 7^th^ passage melanocyte cultures (Figure 1A and 1B). TRP-1 and c-Kit mRNA expressions were determined at every passage by real-time RT-PCR analysis. TRP-1 mRNA values decreased during culturing, exhibiting similar tendency as the protein expression (Figure 1C). This is in line with previous results, showing that TRP-1 is regulated at the transcriptional [22] level. The decrease in TRP-1 mRNA levels from the 4^th^ passage to the 7^th^ passage samples were statistically significant compared to the 1^st^ passage samples. c-Kit mRNA values showed decreasing tendency, but changes in mRNA expressions were not statistically significant (Figure 1D). Values are shown as relative expressions compared to the first passage samples. Averages were calculated from three independent experiments.

### Pigmented, mature melanocytes dedifferentiate in vitro when PMA containing medium is switched to PMA-free Mel-mix

**Figure 2:**
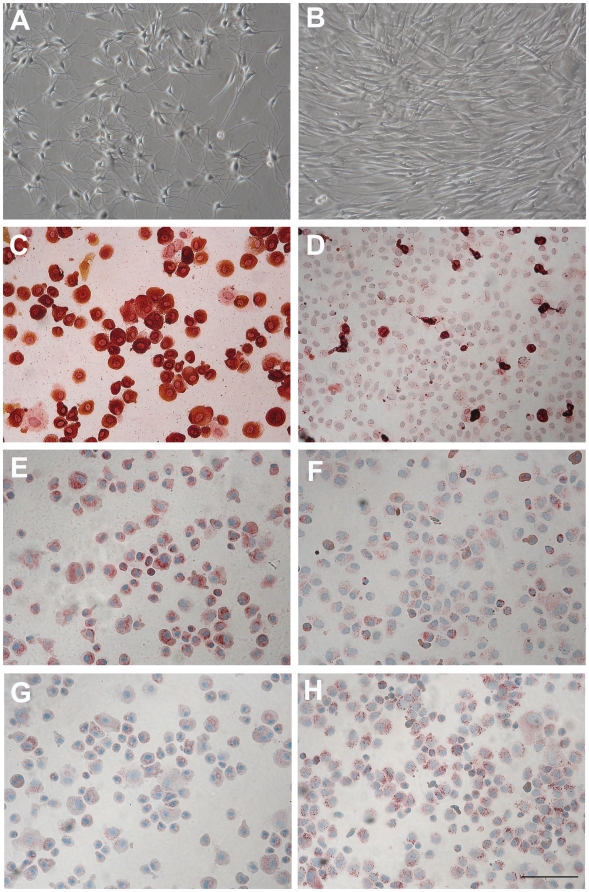
Switching the PMA-containing M254 medium to PMA-free Mel-mix medium results in cell morphology changes and loss of c-Kit and TRP-1 expressions. When melanocyte cultures were initiated in PMA-containing M254 medium, cells were dendritic, and expressed TRP-1 as well as c-Kit proteins (100% of the cells expressed TRP-1 and c-Kit). At week 4, when cells were in 4^th^ passage, half of the cultures were switched to PMA-free Mel-mix medium. Cells in Mel-mix medium became bipolar (B), lost TRP-1 (D) and c-Kit (F), and increased EGFR (H) expressions in two weeks, while melanocytes continuously cultured in M254 medium remained dendritic (A) expressed both TRP-1 (C) and c-Kit (E) proteins, and showed only a week EGFR staining (G). Bar: 100 µm.

In order to study the possible dedifferentiation of melanocytes, normal human adult epidermal melanocytes were cultured in commercially available PMA containing M254 medium for four weeks. These cells showed dendrite-rich morphology, expressed TRP-1 and c-Kit proteins uniformly and EGFR weakly. After four weeks, cultures were split into two; one part was continuously cultured in M254 medium; the other part was switched to Mel-mix medium for the following two weeks. Melanocytes cultured in the PMA containing M254 medium remained dendritic (Figure 2A) and expressed TRP-1 (100% of the cells were positive, Figure 2C) and c-Kit (100% of the cells were positive, Figure 2E) proteins and showed a generally week EGFR staining (only 32% of the cells showed weak positivity, Figure 2G), while cells cultured in Mel-mix for two weeks became bipolar (Figure 2B), their TRP-1 (Figure 2D) and c-Kit (Figure 2F) protein expressions drastically decreased (TRP-1^+^cells: 16%, c-Kit^+^cells: 25%), and their EGFR expression strongly increased (EGFR^+^ cell: 80%) (Figure 2H).

### Cholera toxin and PMA induce dendrite formation, a significant c-Kit, a slight TRP-1 mRNA expressions and the reappearance of c-Kit and TRP-1 proteins in dedifferentiated melanocytes

**Figure 3:**
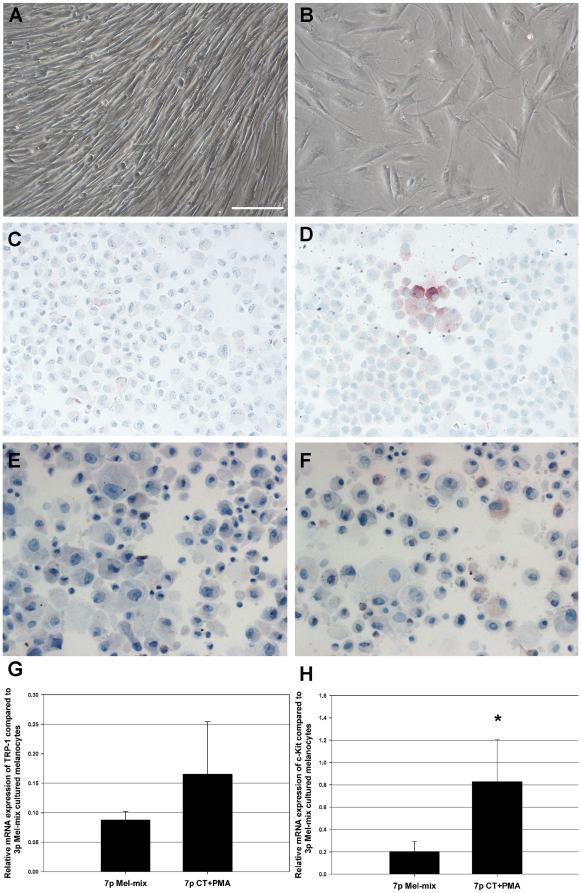
CT and PMA induce dendrite formation, TRP-1 protein and c-Kit mRNA and protein expressions in dedifferentiated melanocytes. Cultured, 7^th^ passage melanocytes showed a uniform, bipolar morphology in Mel-mix medium (A), while treatment of melanocytes with CT and PMA induced dendrite formation (B). CT+PMA induced both TRP-1 (D) and c-Kit (F) protein expressions in melanocytes compared to untreated cultures (C and E) as demonstrated by immunocytochemistry. Bar: 100 µm. At the mRNA level, CT and PMA caused a 2-fold increase in TRP-1 (G) and 4-fold increase in c-Kit (H) expressions compared to untreated controls. Values are shown as relative expressions compared to 3^rd^ passage Mel-mix cultured cells (mean±SD from three independent experiments; *significant at p≤0.05, Univariate ANOVA).

To check the reversibility of the observed dedifferentiation, we added cholera toxin (CT) and phorbol esther (PMA) to the Mel-mix medium, and cultured 7^th^ passage dedifferentiated melanocytes (Figure 3A) for 1 week in this environment. CT+PMA treatment induced dendrite formation in bipolar, dedifferentiated melanocytes (Figure 3B). Immunocytochemical staining demonstrated that TRP-1 protein expression was induced in 7% of the cells (Figure 3D), while c-Kit protein expression was induced in 12% of the dedifferentiated melanocytes (Figure 3F) compared to untreated cultures (TRP-1^+^cells: 0%, c-Kit^+^cells: 0%) (Figure 3C and E).

Real-time RT-PCR was performed to determine the relative expression of TRP-1 and c-Kit mRNA in CT+PMA containing and Mel-mix control cultures. The relative mRNA expression of TRP-1 was on average only 2 times higher in cultures treated with CT+PMA compared to untreated melanocytes grown in Mel-mix medium (Figure 3G). This difference was not statistically significant. The relative mRNA expression of c-Kit was on average 4 times higher in the CT+PMA-treated cultures (Figure 3H). This difference was statistically significant. Values are shown as relative expressions compared to the first passage samples (mean±SD from three independent experiments; *significant at p≤0.05, Univariate ANOVA).

CT and PMA treatment also increased the melanin-content of the cells (Figure S3).

### Melanocytes cultured in Mel-mix medium proliferate more rapidly than cells in PMA containing M254 medium

**Figure 4:**
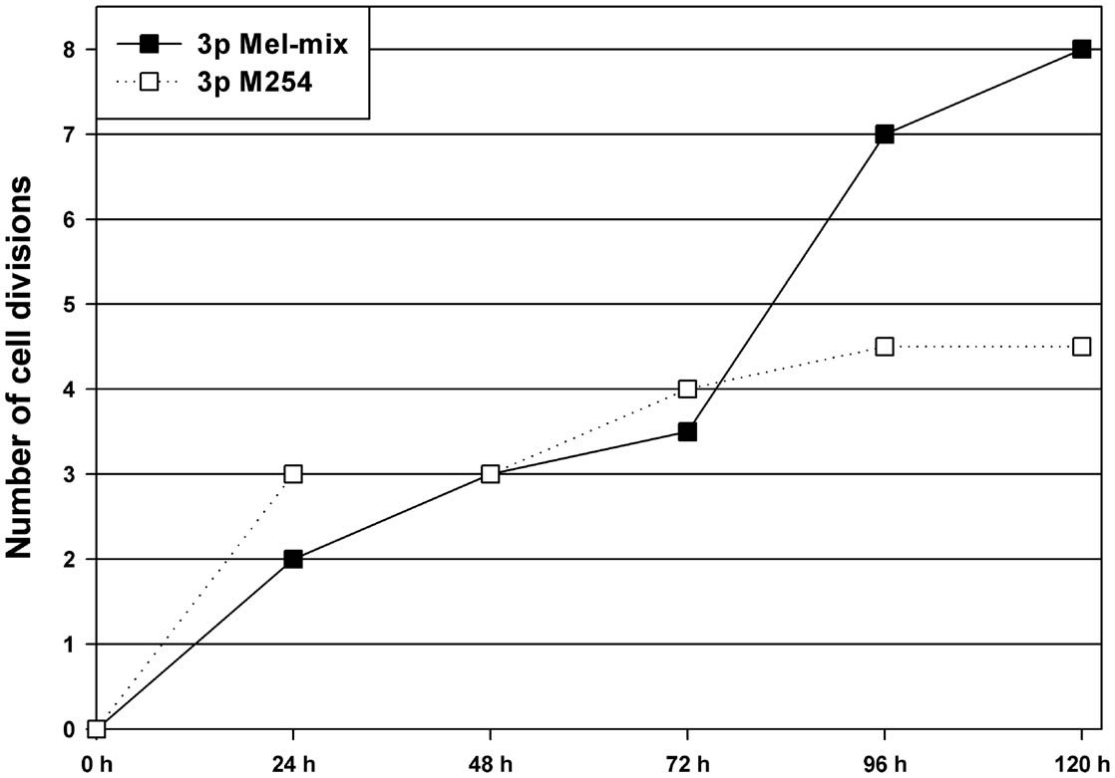
Melanocytes cultured in PMA-free Mel-mix medium proliferate more rapidly than melanocytes cultured in PMA-containing M254 medium. CFSE measurements were performed on melanocytes cultured in Mel-mix and M254 medium. Based on CFSE peak fluorescence intensity halving, cell division numbers for melanocytes cultured in Mel-mix medium (solid line) was 8, while for cells cultured in M254 medium (dashed line) was only 4.5 in the 120-hour experimental period.

To characterize the proliferative capacity of melanocytes cultured in Mel-mix medium, CFSE analysis was performed. Melanocyte culture was initiated from one donor in Mel-mix medium. At the 1^st^ passage, cells were divided into two parts: one part was continuously cultured in Mel-mix, the other part was cultured in PMA containing M254 medium. Both cultures were in 3^rd^ passage when the CFSE analysis was performed. After labeling, samples were collected in every 24 hours for 120 hours and the fluorescence intensity was measured. Cells in both cultures proliferated at a similar rate in the first 72 hours, then melanocytes cultured in Mel-mix medium showed an enhanced rate of division compared to melanocytes cultured in M254 medium. Based on CFSE peak fluorescence intensity halving, the calculated number of cell divisions was only 4.5 for melanocytes cultured in M254 medium, while cells cultured in Mel-mix medium completed 8 dividing cycles in the 120-hour experimental period (Figure 4).

### Senescent cells are equally present in PMA-free and PMA-containing melanocyte cultures

**Figure 5:**
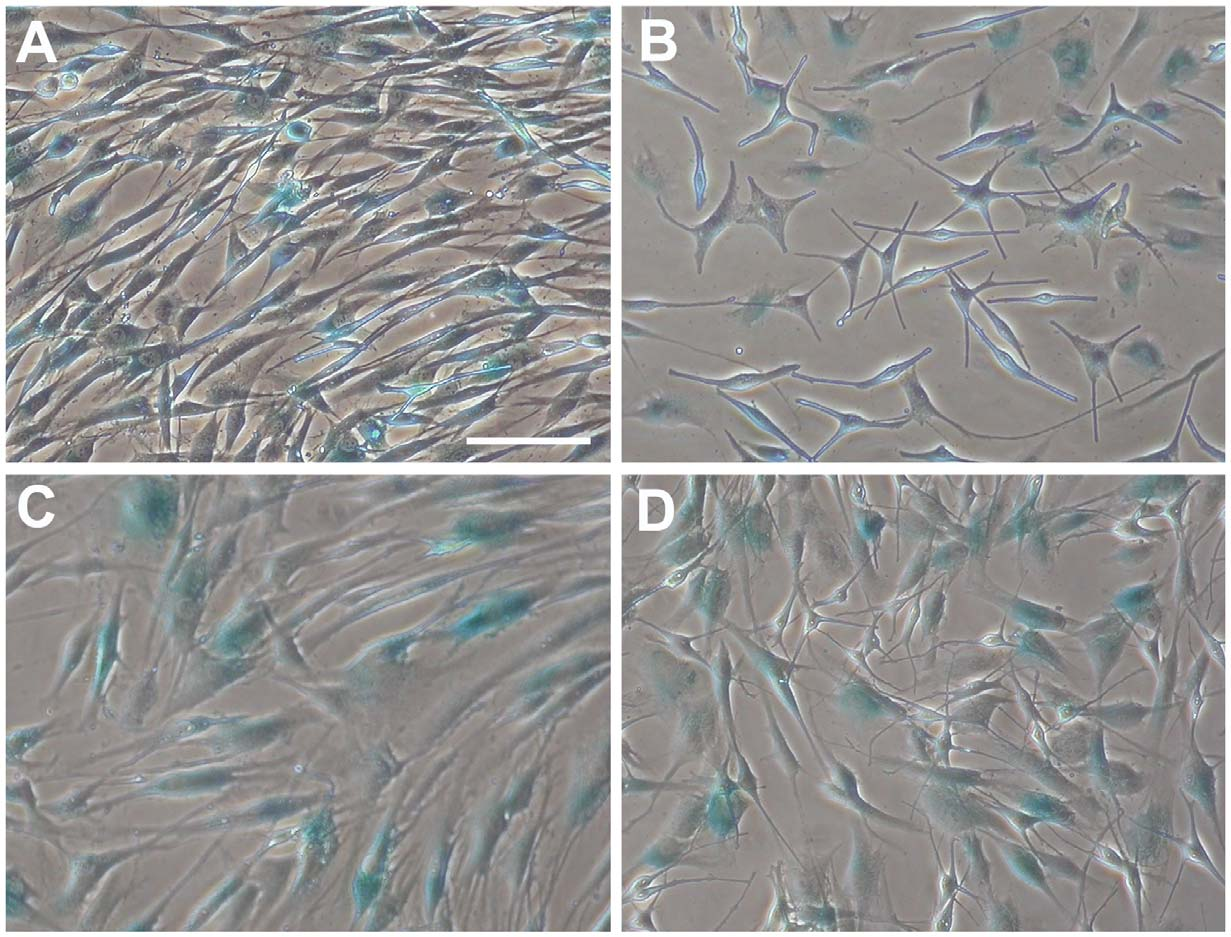
Melanocyte cultures contain about equal number of senescent cells regardless of culture conditions. β-galactosidase assay was performed to detect senescent cells (blue cells) in the cultures. 3^rd^ passage melanocytes cultured in Mel-mix medium (A), 3^rd^ passage melanocytes cultured in M254 medium (B), 8^th^ passage melanocytes cultured in Mel-mix medium (C), 8^th^ passage melanocytes cultured in M254 medium (D). Bar: 100 µm.

Senescence-associated β-galactosidase assay was performed on both types of cultures when cells were in 3^rd^ and 8^th^ passages. Blue colored senescent cells were present in comparable numbers in both types of cultures regardless of the applied media. In 3^rd^ passage cultures 50% of the cells were β-galactosidase^+^ in Mel-mix and 57% in M254, in 8^th^ passage cultures 57% β-galactosidase^+^ cells were in Mel-mix and 48% in M254 (Figure 5). Blue cells appeared flat without dendrites in cultures, irrespective of the used media.

### Direct UVB effect decreases, while indirect, keratinocyte-mediated UVB effect increases the relative mRNA expression of TRP-1 in dedifferentiated melanocytes cultured in Mel-mix

**Figure 6:**
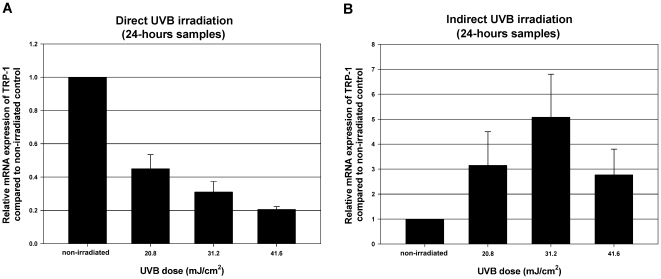
Direct UVB irradiation decreases, while indirect, keratinocyte-mediated UVB effect increases the relative mRNA expressions of TRP-1 in dedifferentiated melanocytes. Dedifferentiated melanocytes cultured in Mel-mix medium were irradiated with 20.8 31.2 and 41.6 mJ/cm^2^ doses of UVB. Direct UVB irradiation decreased TRP-1 mRNA expression in melanocytes (A). Keratinocytes were also irradiated with the same UVB doses. Supernatants were collected from irradiated keratinocytes and autologous melanocytes were treated with them. The “indirect” UVB irradiation caused an increase in TRP-1 mRNA expression (B). Values are shown as relative expressions compared to non-irradiated samples (mean±SEM from two independent experiments).

To study the effect of UVB on melanocyte differentiation, we irradiated 3^rd^ passage melanocytes cultured in Mel-mix medium with 20.8, 31.2 and 41.6 mJ/cm^2^ doses of UVB. Cell viabilities 24 hours after irradiation were: 98±2% at 20.8 mJ/cm^2^, 84±12% at 31.2 mJ/cm^2^ and 77±14% at 41.6 mJ/cm^2^ compared to the viability of non-irradiated control cultures. TRP-1 mRNA expression was used as a marker for melanocyte differentiation. Interestingly, direct UVB irradiation of melanocytes resulted in decreased relative mRNA expression of TRP-1 24 hours after irradiation (Figure 6A). As melanocyte differentiation is also influenced by its *in vivo* environment, we irradiated keratinocytes with the same UVB doses to detect the keratinocyte driven UVB effect on melanocytes. Keratinocyte supernatants were collected 6 and 24 hours after UVB irradiation and third passage cultured autologous melanocytes were treated with these supernatants for 24 hours. Keratinocytes were more sensitive to UVB irradiation in comparison with melanocytes. 24 hours after UVB irradiation 61±11% of keratinocytes were alive at 20.8 mJ/cm^2^ dose, 54±11% at 31.2 mJ/cm^2^ and 34±13% at 41.6 mJ/cm^2^ compared to non-irradiated control cells. Keratinocyte supernatants collected 6 hours after irradiation caused a slight increase in TRP-1 mRNA expression (data not shown), while supernatants collected 24 hours after similar UVB irradiation caused a more pronounced increase in TRP-1 mRNA expression in melanocytes compared to cells that were treated with non-irradiated keratinocyte supernatants. The increase was almost three times higher with supernatants collected from 20.8 and 41.6 mJ/cm^2^ UVB irradiated keratinocytes and more than five times higher with supernatants from 31.2 mJ/cm^2^ UVB irradiated keratinocytes (Figure 6B). Neither indirect nor direct UVB irradiation influenced cell morphology (data not shown). Values are shown as relative expressions compared to non-irradiated samples (mean±SEM from two independent experiments).

### Nestin, a neuronal precursor is expressed by dedifferentiated melanocytes

**Figure 7:**
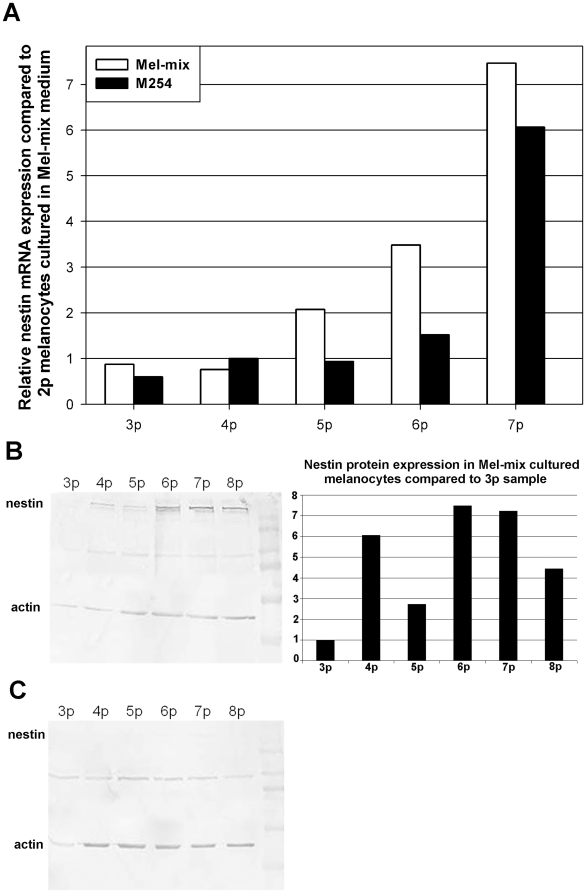
Nestin is strongly expressed in dedifferentiated melanocytes. Although nestin mRNA increased with increasing passage number in all cultured cells this increase was more pronounced in dedifferentiated melanocytes (A). Using a monoclonal antibody, nestin was easily detactable in dedifferentiated cells (B), while in differentiated melanocytes only weak specific bands, not detectable by densitometry, were visible on the blot (C). Densitometry showed increasing expression in dedifferentiated cells during culturing (B).

To further characterize the dedifferentiation state of cultured melanocytes we cultured cells in Mel-mix up to 2^nd^ passage then transferred half the cells into PMA containing M254 medium and left the other half in Mel-mix. We then measured the expression of nestin, a neuronal precursor marker in the different cultures. We were able to detect nestin mRNA in all cultured melanocytes irrespective of culture conditions (Figure 7A). An increase in nestin mRNA expression was observed in cells as they reached higher passages. The increase of nestin mRNA was more pronounced in dedifferentiated melanocytes cultured in the PMA-free Mel-mix medium (Figure 7A, Figure S4). Values are shown as relative expressions compared to the 2^nd^ passage sample. On Western blot, although a faint nestin specific band was visible in all samples (Figure 7B and C), densitometry could not detect bands from samples of PMA containing M254 cultured melanocytes. A gradual increase in nestin protein expression was detected only in Mel-mix cultured, *in vitro* dedifferentiated cells (Figure 7B).

## Discussion

The traditionally used media for melanocyte culturing contain specific mitogens, such as PMA, CT and IBMX. Melanocytes cultured in media that contains PMA, CT or IBMX show the phenotype of fully differentiated melanocytes. Differentiated melanocytes are characterized by melanin production due to the activities of tyrosinase, TRP-1 and TRP-2; by numerous mature melanosomes and well-developed dendrites [3]. Melanocytes cultured without PMA, CT or IBMX show pigmentation loss and decreased dendrite formation [20], [23], [24].

In melanocyte development, c-Kit plays a critical role in a number of cellular activities, including differentiation. The expression of c-Kit is considered a key step in pigment cell development, Kit+ nonmelanotic cells in the skin are putative melanocyte precursors [25]. Activation of c-Kit by stem cell factor results in Mitf phosphorilation [8]. Microphtalmia associated transcription factor (Mitf) determines the melanocyte fate of multipotent neural crest cells partially by its transcriptional and lineage-specific regulation of three major pigment enzymes, tyrosinase, TRP-1 and TRP-2 [26]. There is also evidence that c-Kit, tyrosinase and TRP-1 gene expression are coordinated in melanocytes [27]. The melanocytes that we harvested from the adult human skin uniformly stained for c-Kit and TRP-1, if they were cultured in PMA containing M254 medium. These cells showed characteristic dendritic morphology and pigment production. Among cells that were grown in Mel-mix medium without PMA, c-Kit expression appeared in fewer cells, and when c-Kit positive cells from PMA containing medium were switched into Mel-mix, the expression of c-Kit protein in the cells decreased dramatically. In the Mel-mix culture, without PMA, cells showed a uniform positivity for TRP-1 in early cultures, whereas in older cultures, we could not detect TRP-1 protein expression in the cells. Similarly TRP-1 positive cells grown in PMA containing medium lost their TRP-1 expression after changing their culture environment to PMA-free Mel-mix. In Mel-mix medium, TRP-1 and c-Kit mRNA expression also showed a decreasing trend. The decrease in c-Kit mRNA level was not significant, less dramatic than the disappearance of c-Kit protein expression in the cells. These data indicate that differentiation is a reversible process in epidermal melanocytes. Depending on the culture conditions, cells can revert to a less differentiated form. In human skin, c-Kit positive cells consist of two populations, one expresses TRP-1 and the other does not [11]. It has been proposed that the c-Kit+ TRP-1- cell population, also characterized by strong BCL-2 expression, represents less differentiated melanocytes. If differentiated melanocytes die, these cells could serve as a source to repopulate the skin. It is also possible that under certain conditions, when the need for melanization subsides, cells could revert to a less differentiated form [11].

*In vitro* studies have shown that TPA (PMA) induces neural crest cell differentiation into melanocytes and stimulates proliferation and differentiation of normal melanocytes [4], [28]. Similarly, in our culture system, the addition of cholera toxin and PMA to the Mel-mix medium resulted in dendrite formation and induction of c-Kit mRNA and protein expression in the *in vitro* dedifferentiated melanocytes. The amount of TRP-1 mRNA also showed increasing tendency and in a few cells, TRP-1 protein expression reappeared. The fact that c-Kit expression precedes TRP-1 expression is expected, because c-Kit signaling is essential for the transcription of TRP-1 [26].

It has been reported that withdrawal of cAMP inducers (CT+IBMX) from the medium in melanocyte cultures causes cells to become senescent [29]. Although, our culture medium lacks these mitogens, melanocytes proliferate rapidly in this medium. In fact, melanocyte proliferation was higher in Mel-mix medium than in PMA-containing melanocyte growth medium. This high proliferative rate of Mel-mix cultured adult melanocytes typically lasts until the 10^th^ passage, then proliferation slows, and by about the 15^th^ passage, cell growth arrests. We found no difference in senescent cell numbers between cultures of Mel-mix and PMA-containing M254 medium.

We used our culture system to study the direct and indirect effects of UVB on melanocyte differentiation/pigmentation. TRP-1 mRNA expression was used as a marker to determine the effect of UVB on melanocyte differentiation/pigmentation. Human TRP-1 has tyrosine hydroxylase activity, it has a role in processing and stabilizing the enzymatic activity of tyrosinase, thereby it takes part in maintaining the structural integrity of melanosomes (for a review see [30], [31]). Data indicate that UVB can influence the expression of TRP-1 [32], [33]. Repeated UVB irradiation induced TRP-1 expression in melanocyte-keratinocyte co-culture [34]. In our experiments, direct UVB exposure caused a decrease in TRP-1 mRNA expression in melanocytes. Similar results have already been reported: in the absence of cAMP inducers, UVB radiation inhibited, rather than stimulated, melanogenesis [35]. On the other hand, the indirect effect of UVB irradiation on melanocytes, exerted through keratinocyte soluble factors, resulted in TRP-1 mRNA induction in the cells. The most likely keratinocyte-derived factors which may be responsible for this TRP-1 mRNA induction are α-melanocyte stimulating hormone (α-MSH) and endothelin-1 (ET-1). It is well documented that UVB induces the production of both factors [36]. Our results are in line with numerous data indicating that melanocyte differentiation and melanogenesis are influenced by tissue environment, in which keratinocytes are key (for a review see [3]).

Melanocytes arise from the neural crest, a pluripotent structure of the vertebrate embryo. In addition to melanocytes and many other cell types, neural crest is also the source of neurons and glia of the peripherial nervous system. During the segregation of cell lineages derived from the neural crest, multipotent neural-melanocytic progenitors and bipotent glial-melanocytic precursors are generated. From the bipotent glial-melanocytic precursors, melanoblasts and melanocytes originate [37]. Cell differentiation is not unidirectional; under certain stimuli *in vitro* or during regeneration, differentiated cells may recover properties of immature cells [38]. It has been shown that neural crest-derived pigment cells from quail embryo could dedifferentiate/transdifferentiate into glia through a glial-melanocytic progenitor, if treated with endothelin-3 (ET-3) [39]. A recent study in mice identified growing nerves projecting throughout the body as progenitor niche containing Schwann cell precursors from which large numbers of melanocytes originate [26]. It is known that cutaneous melanocytes share many signaling molecules with neurons, and *in vitro* melanocyte cultures have already been proposed to be used as model system to study Alzheimer's disease [40]–[42].

To characterize the stage of dedifferentiation of adult melanocytes cultured in Mel-mix medium, we examined the expression of nestin, a “neural stem/progenitor cell” marker in the cells. Nestin first appears in cells of nervous tissue formed during the embryonic period of ontogenesis [43]. Upon differentiation, nestin is down-regulated, but its re-expression has been demonstrated in a variety of primary central nervous system (CNS) tumors, in injured tissues of CNS, and in melanoma. It has been suggested that nestin was an indicator of cell-dedifferentiation in melanocytes, as nestin protein was found to be abundant in melanoma [44], [45]. In a recent study, nestin expression was detected in cultured normal human foreskin melanocytes [46]. We found strong nestin expression both at the mRNA and protein levels in dedifferentiated melanocytes. Differentiated cells expressed less nestin mRNA and almost undetectable nestin protein. We have preliminary results showing that another neural precursor marker, the translocator protein (TSPO) is also expressed in both type of cultured melanocytes at the mRNA level (data not shown). Translocator protein 18 kDa, the peripheral benzodiazepine receptor by its earlier name, is a mitochondrial membrane protein associated with the mitochondrial permeability pore. In the healthy adult brain, TSPO expression is restricted to glial cells. However, in developing or damaged neural regions, TSPO appears in differentiating/regenerating neurons similar to nestin. TSPO mRNA and protein, while missing from mature neurons, are present in neural stem cells and also in postmitotic neuronal precursors [47]. Further studies are needed to clarify how abundant TSPO expression is in our dedifferentiated melanocytes and how it translates to protein expression.

Earlier we have shown that normal human adult epidermal melanocytes expressed EGFR and responded to EGF [20]. It is known that EGF and its receptor, EGFR have an important role in neuronal differentiation [48], [49]. Here we show that EGFR expression is induced in dedifferentiated melanocytes.

Melanocyte differentiation is usually studied on quail embryo skin and on mouse neural crest cell lines. Until now, there has been no suitable human model system for studying melanocyte differentiation. Our human melanocyte culture can serve as a model system to study melanocyte proliferation/differentiation, and melanoma development. Besides that, melanocytes cultured in chemical mitogen-free medium are applicable in the therapy of pigmentation-associated disorders, like vitiligo. Cells expanded *in vitro* in Mel-mix medium supplemented with autologous human serum instead of FBS and BPE allow for autologous transplantation of cultured melanocytes in vitiligo patients in early passages when their pigmentation is not lost [20]. Further work is needed to examine the ability of neuronal transdifferentiaton of these *in vitro* cultured melanocytes. If these cells were able to transdifferentiate into neuronal precursors, they could also be considered as potential therapeutic tools for different neurodegenerative diseases.

## Supporting Information

Figure S1 **COL1A2 gene is only expressed in the dermis, not in the epidermis after dispase digestion.** Melanocytes from adult human skin were separated using dispase to split the epidermis from the dermis. To prove that dispase treatment indeed separates the epidermis from the dermis without fibroblast contamination we performed real-time PCR with primers specific for the COL1A2 gene. We could detect COL1A2 gene expression only in the dermal samples gained after dispase digestion of the skin (see figure below, n = 3, bar shows mean ± SEM).
(TIF)

Figure S2 **Cultured melanocytes can be separated from keratinocytes due to their different attachment characteristics.** After a short trypsinization, melanocytes release from culture dish 2–3 minutes earlier than keratinocytes, thus enabling separation of the two cell populations. Trypsinization time: a: 0 min, b: 2 min, c: 3 min, d: 6 min. Magnification: 200x.
(TIF)

Figure S3 **CT and PMA treatment increased the melanin-content of melanocytes.** Mel-mix cultured melanocytes in 7^th^ passage were switched into M254 medium or treated with 10 nM cholera toxin, 10 ng/ml PMA and with both 10 nM cholera toxin and 10 ng/ml PMA for one week. An individual melanocyte in 7^th^ passage culture growing in PMA-free Mel-mix medium contained 39 pg of melanin. Switching the PMA-free Mel-mix medium to PMA-containing M254 medium increased the melanin content in the cells to 58 pg melanin. PMA treatment of Mel-mix cultured melanocytes raised pigment content to 68 pg/cell. Cholera toxin caused only a slight increase in melanin-production, melanocytes in this culture contained 47 pg/cell melanin. Simultaneous addition of CT and PMA showed similar result than CT treatment alone (48 pg/cell). Results are from one experiment.
(TIF)

Figure S4 **Nestin mRNA expression increased with dedifferentiation.** To further prove that nestin mRNA expression was higher in dedifferentiated melanocytes we performed real-time RT-PCR from multiple Mel-mix cultured cells. As the cells dedifferentiated in culture -going through passages from 1-3-7 in Mel-mix medium- their nestin mRNA expressions increased. Values are shown as relative expressions compared to one of the first passage samples. Averages were calculated from three independent experiments.
(TIF)
