# Supplementary figures and images for: In Vitro Dedifferentiation of Melanocytes from Adult Epidermis
Source: PLoS One. 2011 Feb 23;6(2):e17197. doi: 10.1371/journal.pone.0017197 (PMC3044174; doi:10.1371/journal.pone.0017197)

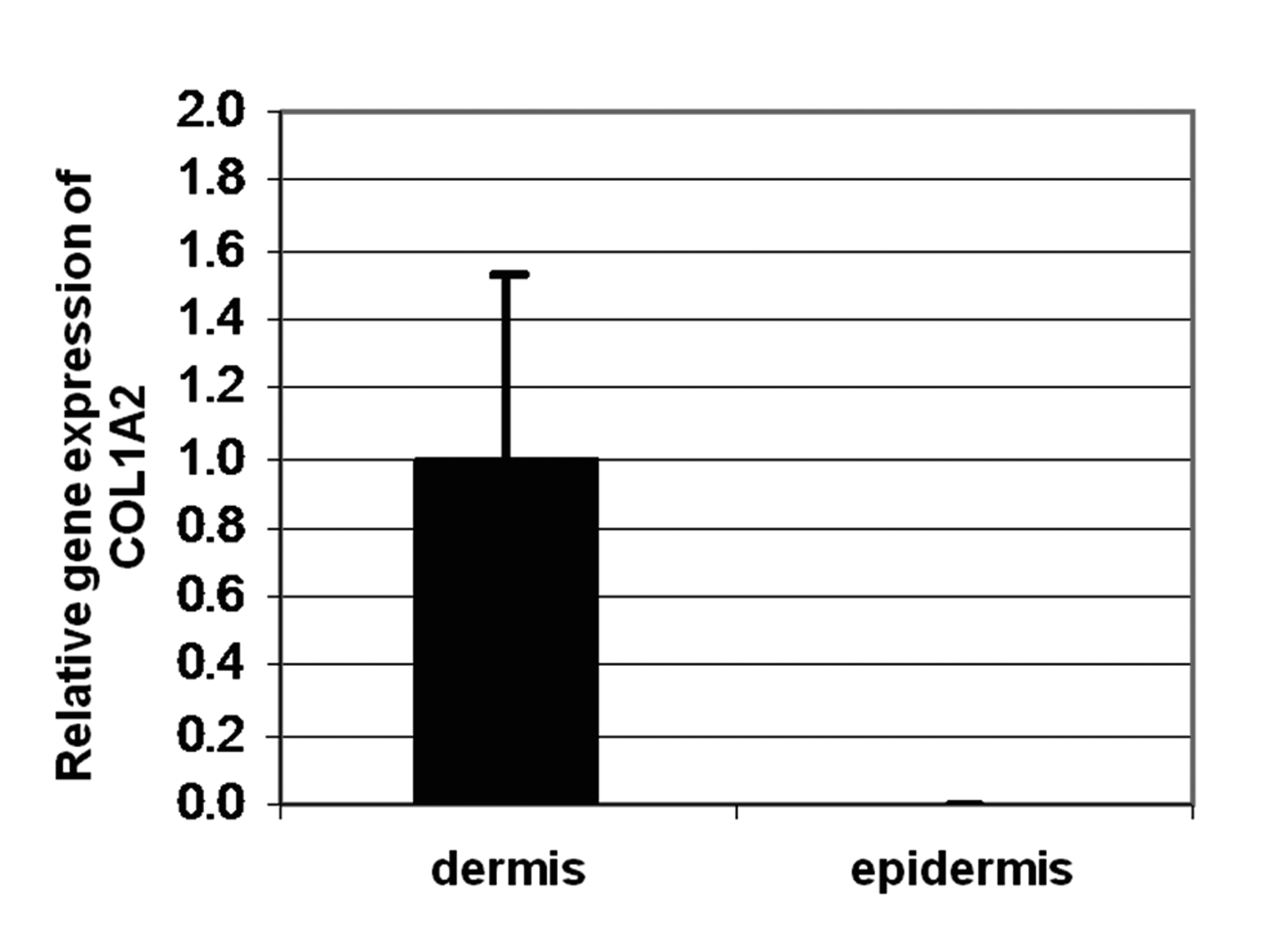

Supplement: Figure S1 — COL1A2 gene is only expressed in the dermis, not in the epidermis after dispase digestion. Melanocytes from adult human skin were separated using dispase to split the epidermis from the dermis. To prove that dispase treatment indeed separates the epidermis from the dermis without fibroblast contamination we performed real-time PCR with primers specific for the COL1A2 gene. We could detect COL1A2 gene expression only in the dermal samples gained after dispase digestion of the skin (see figure below, n = 3, bar shows mean ± SEM). (TIF) [file pone.0017197.s001.tif]

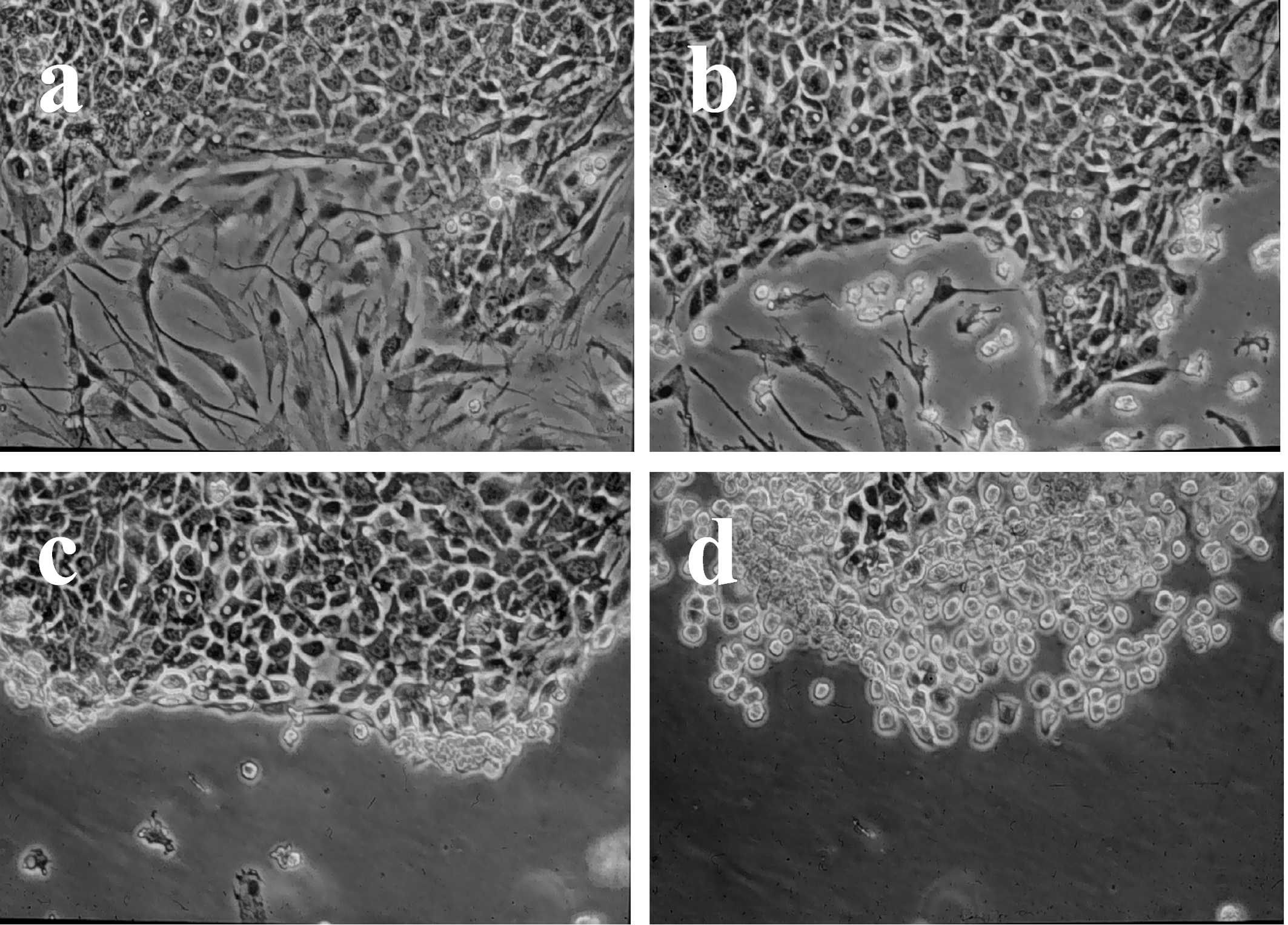

Supplement: Figure S2 — Cultured melanocytes can be separated from keratinocytes due to their different attachment characteristics. After a short trypsinization, melanocytes release from culture dish 2–3 minutes earlier than keratinocytes, thus enabling separation of the two cell populations. Trypsinization time: a: 0 min, b: 2 min, c: 3 min, d: 6 min. Magnification: 200x. (TIF) [file pone.0017197.s002.tif]

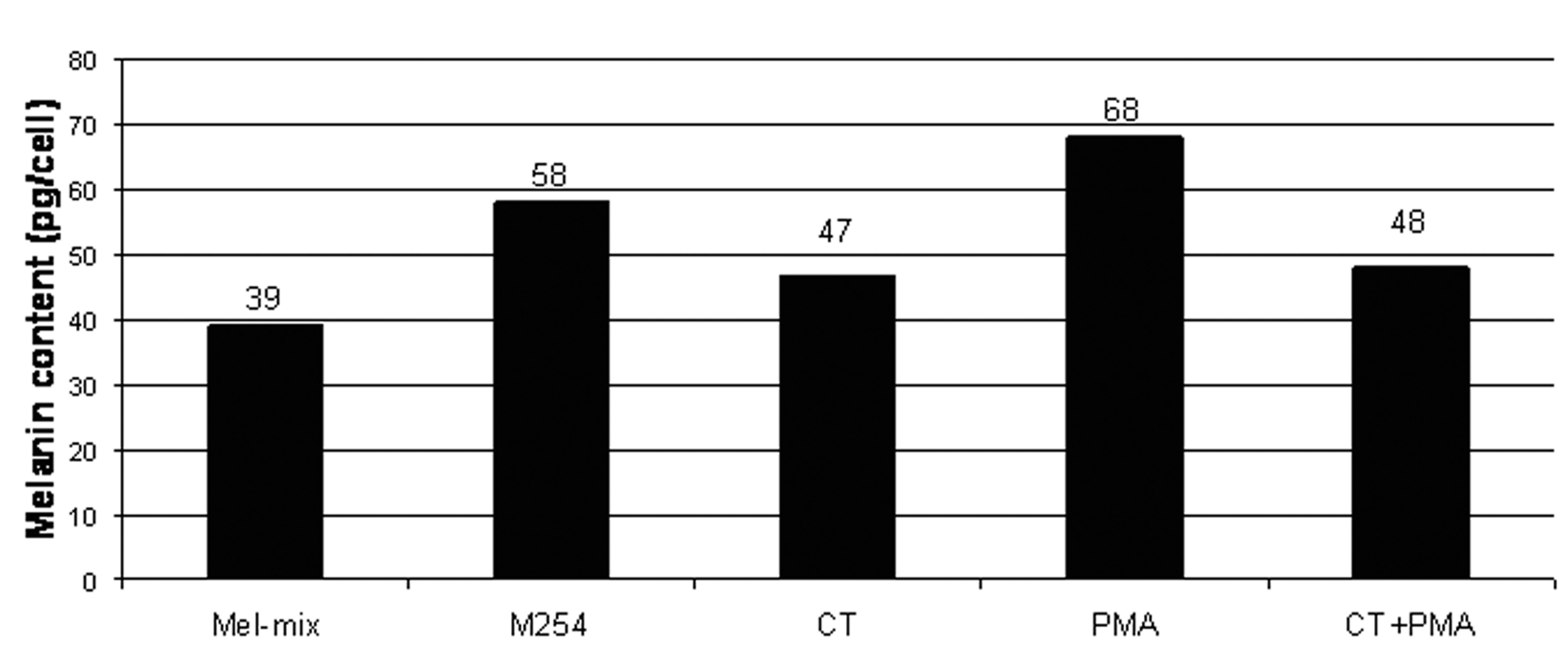

Supplement: Figure S3 — CT and PMA treatment increased the melanin-content of melanocytes. Mel-mix cultured melanocytes in 7th passage were switched into M254 medium or treated with 10 nM cholera toxin, 10 ng/ml PMA and with both 10 nM cholera toxin and 10 ng/ml PMA for one week. An individual melanocyte in 7th passage culture growing in PMA-free Mel-mix medium contained 39 pg of melanin. Switching the PMA-free Mel-mix medium to PMA-containing M254 medium increased the melanin content in the cells to 58 pg melanin. PMA treatment of Mel-mix cultured melanocytes raised pigment content to 68 pg/cell. Cholera toxin caused only a slight increase in melanin-production, melanocytes in this culture contained 47 pg/cell melanin. Simultaneous addition of CT and PMA showed similar result than CT treatment alone (48 pg/cell). Results are from one experiment. (TIF) [file pone.0017197.s003.tif]

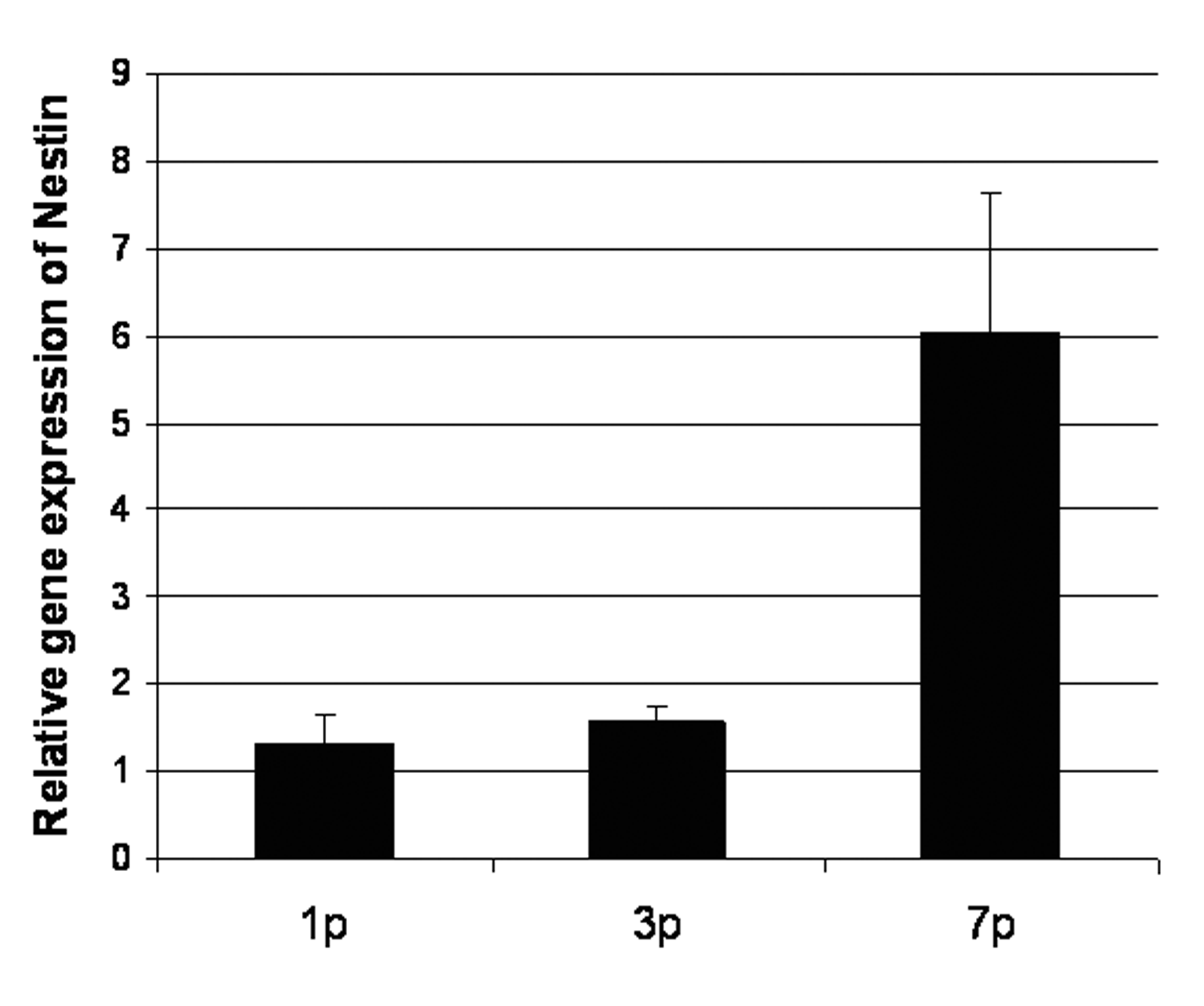

Supplement: Figure S4 — Nestin mRNA expression increased with dedifferentiation. To further prove that nestin mRNA expression was higher in dedifferentiated melanocytes we performed real-time RT-PCR from multiple Mel-mix cultured cells. As the cells dedifferentiated in culture -going through passages from 1-3-7 in Mel-mix medium- their nestin mRNA expressions increased. Values are shown as relative expressions compared to one of the first passage samples. Averages were calculated from three independent experiments. (TIF) [file pone.0017197.s004.tif]
